# Characterisation of enterovirus 71 replication kinetics in human colorectal cell line, HT29

**DOI:** 10.1186/2193-1801-2-267

**Published:** 2013-06-18

**Authors:** Yan Long Edmund Lui, Peter Timms, Louise Marie Hafner, Tuan Lin Tan, Kian Hwa Tan, Eng Lee Tan

**Affiliations:** Centre for Biomedical and Life Sciences, Singapore Polytechnic, Singapore, Singapore; School of Biomedical Sciences, Faculty of Health, Queensland University of Technology, Brisbane, Australia; Institute of Health and Biomedical Innovation, Queensland University of Technology, Brisbane, Australia; School of Chemical and Life Sciences, Singapore Polytechnic, Singapore, Singapore; Department of Paediatrics, University Children’s Medical Institute, National University Hospital, Singapore, Singapore

**Keywords:** Hand, Foot and mouth disease, Enterovirus 71, Virus replication kinetics, Colorectal cell

## Abstract

Hand, Foot and Mouth Disease (HFMD), a contagious viral disease that commonly affects infants and children with blisters and flu like symptoms, is caused by a group of enteroviruses such as Enterovirus 71 (EV71) and coxsackievirus A16 (CA16**)**. However some HFMD caused by EV71 may further develop into severe neurological complications such as encephalitis and meningitis. The route of transmission was postulated that the virus transmit from one person to another through direct contact of vesicular fluid or droplet from the infected or via faecal-oral route. To this end, this study utilised a human colorectal adenocarcinoma cell line (HT29) with epithelioid morphology as an *in vitro* model for the investigation of EV71 replication kinetics. Using qPCR, viral RNA was first detected in HT29 cells as early as 12 h post infection (hpi) while viral protein was first detected at 48 hpi. A significant change in HT29 cells’ morphology was also observed after 48 hpi. Furthermore HT29 cell viability also significantly decreased at 72 hpi. Together, data from this study demonstrated that co-culture of HT29 with EV71 is a useful *in vitro* model to study the pathogenesis of EV71.

**Electronic supplementary material:**

The online version of this article (doi:10.1186/2193-1801-2-267) contains supplementary material, which is available to authorized users.

## Background

Hand, Foot and Mouth Disease (HFMD), a contagious viral disease that commonly affects infants and children, are caused by a group of enteroviruses such as Enterovirus 71 (EV71) and coxsackievirus A16 (CA16**)** ( Brown et al. 1999 ; Cardosa et al. 2003 ; Lee et al. 2009 ; Prager et al. 2003 ). This self-limiting disease is characterised by fever, rashes, poor appetite and multiple ulcers in mouth ( Brown et al. 1999 ; Cardosa et al. 2003 ; Lee et al. 2009 ; Prager et al. 2003 ). However, patients infected with EV71 may further develop severe neurological complication such as aseptic meningitis and brainstem/cerebellar encephalitis ( Lee and Chang 2010 ; Lee et al. 2009 ; Singh et al. 2002 ; Solomon et al. 2010 ; McMinn 2002 ).

EV71, a member from the Enterovirus genus of the Picornaviridae family, is a non-enveloped, positive sense, single stranded, RNA virus with genomic RNA of approximately 7400 bp in length ( Lee and Chang 2010 ; Oberste et al. 1999a , b ). EV71 was first isolated from HFMD patients with central nervous system disease in 1969 ( Schmidt et al. 1974 ). Large fatal EV71 outbreaks of HFMD first appeared in Bulgaria in 1975, and disease outbreaks were subsequently identified in Hungary in 1978 and re-emerged in Malaysia in 1997 and Taiwan in 1998 ( Chumakov et al. 1979 ; Nagy et al. 1982 ; Ho et al. 1999 ; Lu et al. 2002 ; Solomon et al. 2010 ). HFMD epidemics and pandemics have been periodically reported worldwide with outbreaks occurring every two to three years in countries including Australia, China, Taiwan, Japan, Korea, Malaysia, Vietnam, Thailand and Singapore ( Schmidt et al. 1974 ; Chumakov et al. 1979 ; McMinn et al. 2001 ; Huang et al. 2012 ). Although the consistent presences and outbreaks of HFMD push for an urgent need to develop a vaccine or antiviral therapies against enteroviruses ( Pourianfar et al. 2012 ; McMinn et al. 2001 ; Tan et al. [Bibr CR43][Bibr CR44] ; Tan et al. 2010 ). Currently, there are no available antiviral therapies or vaccines approved by the United States Food and Drug Administration (FDA) to prevent HFMD infections ( Li et al. 2007 ).

The route of transmission of EV71 was postulated to happen via direct contact of vesicular fluid or droplet from the infected or via faecal-oral route ( Wong et al. 2010 ; Lee et al. 2009 ; Liu et al. 2000 ; Brown et al. 1999 ). EV71 was shown to replicate within the gastrointestinal tract, bypass the gut barrier and infect into the skeletal muscle cell before entering into the bloodstream and the central nervous system ( Wong et al. 2010 ; Lee et al. 2009 ; Liu et al. 2000 ; Brown et al. 1999 ). Indeed various studies had shown in *in vivo* model such as mouse that the intestine was the initial site of infection for EV71 infection with the muscle cells responsible for persistent infection supporting efficient virus replication ( Chen et al. 2007 ; Chen et al. 2004 ; Khong et al. 2012 ). Therefore we hypothesised that it is relevant to study EV71 using an *in vitro* model of a human gastrointestinal cell type origin during the initial stages of infection.

In this study, we used a human colorectal adenocarcinoma cell line (HT29) with epithelioid morphology as an *in vitro* model for the investigation of EV71 replication kinetics. To characterise the virus replication in HT29 cells, the viral VP1 RNA and protein were monitored using qPCR and western blot respectively. In addition, the cell viability of HT29 to EV71 infection was monitored throughout the time course of 72 hours posts infection (hpi).

## Results

This study aims to characterise the viral replication in an *in vitro* model of HFMD for EV71 pathogenesis study. To this end a human colorectal adenocarcinoma cell line (HT29) with epithelioid morphology was infected with EV71 over the time course of 72 h.

### Cytopathic effects and cell viability of HT29 cells during EV71 infection

**Figure 1:**
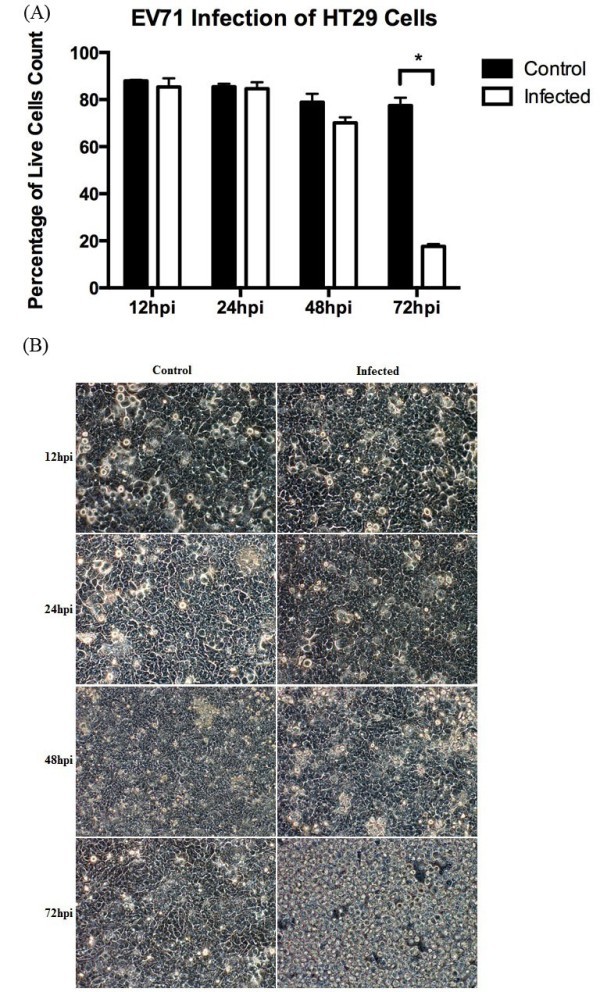
**Cell viability of HT29 cells following EV71 infection.** Confluent HT29 cells were infected with or without EV71 (MOI of 1). (**A**) HT29 cell harvested at different time points and cell viability assessed using vital dye trypan blue. (n = 3, * = *p* values of < 0.05) (**B**) Micrograph of confluent HT29 cell cultures were taken at a magnification of 20× at different time points for 72 h.

There are no changes between control and infected cells at 12 hpi, 24 hpi and 48 hpi (Figure 1). However at 72 hpi there was a statistically significant decrease of approximately 60% of the infected cells (17.6%) as compared to control (77.5%). There are no apparent change in morphology between infected and control cells at 12 hpi and 24 hpi. However, at 48 hpi and 72 hpi, cells were observed to lose its adherence and round up which suggest cells are under stress conditions (Figure 1) with cytopathic effect observed. At 72 hpi, in comparison with the control and infected cells, there was a higher number of floating cells in the media which correlate to the decrease in live cells count (Figure 1).

### Viral kinetics of EV71 replication

**Figure 2:**
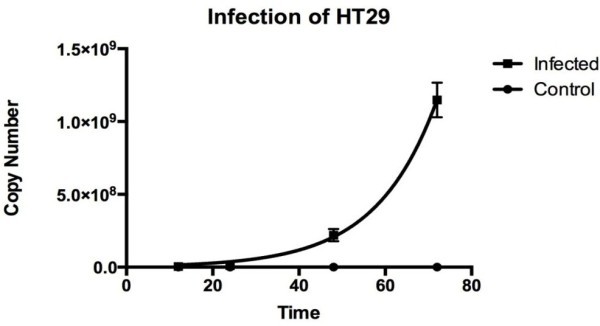
**Kinetics of EV71 replication in HT29 cells.** Confluent HT29 cells were infected with or without EV71 (MOI of 1). Total intracellular RNA were harvested at various time points, converted to cDNA and measured by quantitative real time polymerase chain reaction (qRT-PCR) with primers specific to viral VP1. (n = 3, * = *p* values of < 0.05) As observed, there was an increase in viral copy number through increasingly time points.

To further study EV71 replication kinetics in HT29 cells, the viral RNA and protein synthesis was monitored over 72 h. Using established protocols, the kinetics of EV71 RNA synthesis in infected HT29 cells were examined quantitatively using qPCR at various time points (12 hr, 24 hr, 48 hr and 72 hr) ( Tan et al. 2008b ). Control cells show no viral RNA presents (Figure 2). There was an expected exponential increase in viral RNA copy number of the infected cells as measured by qPCR. Viral RNA was first detected at 12 hpi. Approximately 5 million virus copy number was detected at 12 hpi, which then doubles at 24 hpi to approximately 10 million virus copy number. Subsequently at 48 hpi, it increases 20 folds to approximately 200 million virus copy number. Finally at 72 hpi, it increases 5 folds to 1000 million virus copy number. The viral copy number quantitated had exponential increase at every time points except 72 hpi where there was only 5 folds increase in virus copy number (Figure 2).

### Viral kinetics of EV71 VP1 protein synthesis

**Figure 3:**
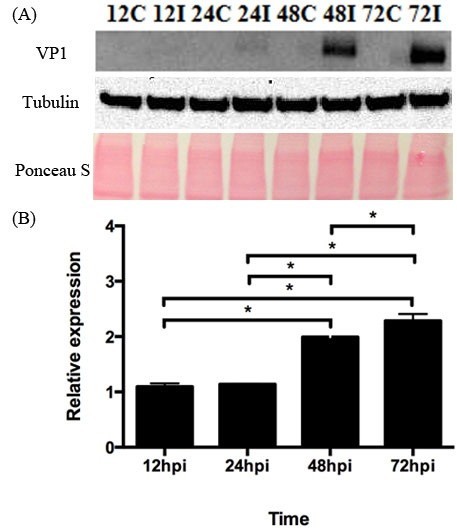
**Kinetics of EV71 VP1 protein synthesis in HT29 cells.** Confluent HT29 cells were infected with or without EV71 (MOI of 1). Total intracellular protein were harvested at various time points and measured by western blot captured using Quantity One software. (n = 3, * = *p* values of < 0.05) The results were then analysed using Image J. As observed, it trends the results we observed by viral RNA amplification. (**A**) Intracellular viral VP1 measured by Western blotting with Ponceau S staining control of membrane (**B**) Relative expression of viral VP1 protein.

In addition to the detection of viral RNA, the kinetics of EV71 VP1 protein synthesis in infected HT29 cells was monitored using western blot specific to EV71 (Figure 3). Relative expression of EV71 VP1 protein of infected and control cells were analysed using Image J and statistical significant amount of virus protein were only detected at 48 hpi and 72 hpi.

### Cellular receptor SCARB2 is present in both HT29 and RD cells

**Figure 4:**
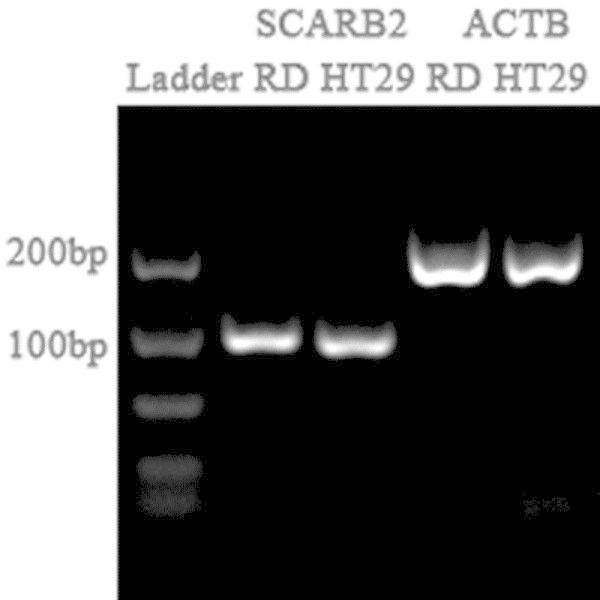
**Expression SCARB2 in RD and HT29 cells.** Total intracellular RNA were harvested from RD and HT29 cells, converted to cDNA and measured by quantitative real time polymerase chain reaction (qRT-PCR) with primers specific to SCARB2 and ACT. Primers for SCARB2 and ACT were designed to span exon-exon boundaries to give a single PCR product of 89 bp and 198 bp respectively. The presence of SCARB2 in HT29 further supports HT29 cells as a viable *in vitro* model to study EV71 pathogenesis.

Receptor binding is an essential and vital process during virus infection of the cells ( Patel and Bergelson 2009 ; Yamayoshi et al. 2012 ). Cellular receptor plays an important role in the pathogenicity of viruses particularly during the internalisation of virus during infection. As such we quantified the relative expression of a functional receptor, Scavenger receptor class B, member 2 (SCARB2) based on the formula: 2(^Ct of gene-Ct of ACT^). Primers for SCARB2 and ACT were designed to span exon-exon boundaries to give a single PCR product of 89 bp and 198 bp respectively (Figure 4). HT29 was found to express SCARB2 at the expression level of approximately 20.2 as compared with RD cells of approximately 26.9. The presence of SCARB2 in HT29 further supports HT29 cells as a viable *in vitro* model to study EV71 pathogenesis.

## Discussion

Viral replication kinetics plays an important role in the understanding of virus pathogenesis ( Baccam et al. 2006 ; Chang et al. 2006 ; Major et al. 2004 ). The kinetics of various viruses such as hepatitis C, Nipah virus and influenza virus have been reported in various studies and have provided valuable information particularly in response to antiviral therapeutics which aid in the understanding the host pathogen interaction ( Baccam et al. 2006 ; Chang et al. 2006 ; Major et al. 2004 ). In comparison, the only information reported for EV71 virus kinetics was by Lu et al. ( 2011 ) who demonstrated EV71 proliferation in rhabdomyosarcoma (RD) cells, of muscle cells origin as an *in vitro* model ( Lu et al. 2011 ). In a clinical context, this may not be a good representative model during EV71 infection of a human host. Various studies have demonstrated in animal model that the gastrointestinal tract such as the intestine was the first site for EV71 proliferation ( Chen et al. 2007 ; Chen et al. 2004 ; Khong et al. 2012 ). Furthermore, Khong et al. ( 2012 ) reported that mice that were administrated with EV71 via the intraperitoneal route exhibits 100% mortality as compared to mice that were administrated with EV71 via the oral route which shows 10-30% mortality ( Khong et al. 2012 ). Interestingly, the mortality result of mice administrated with EV71 via the oral route (gastrointestinal tract) reported by Khong et al. ( 2012 ) corresponded to the percentage of EV71 infected human patient with central nervous system complications. This suggests the relevant of studying EV71 in colorectal cell line (oral route), HT29 as an *in vitro* model ( Ooi et al. 2010 ; Ooi et al. 2009 ; Ooi et al. 2007 ).

In this study, we have demonstrated that upon EV71 infection in human epithelial colorectal cell line (HT29), significant cell death only occurs at 72 hpi. This was varies from the rhabdomyosarcoma (RD) cells previously reported by Lu et al. ( 2011 ) where most cell death occurs within 24 hpi. Similarly Chen et al. ( 2007 ) and Khong et al. ( 2012 ) proposed that skeletal muscle cells such as RD cells are more effective in supporting viral replication which allow persistent enterovirus infection to represent viral source of entry into the central nervous system (CNS) ( Chen et al. 2007 ; Khong et al. 2012 ). In comparison with EV71 RNA synthesis, RNA was first detected at 12 hpi. Viral protein synthesis was only observed at a later stage during the infection possibly due to translational time required. Lu et al. ( 2011 ) showed a similar trend that virus RNA was first detected at 3 hpi while virus protein was observed at 6 hpi. We reckon that such differences could be due to the fact that it takes three times longer for the virus to kill HT29 cells in comparison with RD cells Additional file 1: Table S1.

Receptor binding is an essential and vital process during virus infection of the cells ( Patel and Bergelson 2009 ; Tan et al. 2013 ; Yamayoshi et al. 2012 ). Cellular receptors therefore play an important role in the pathogenicity of viruses. Notwithstanding, viruses have been found to utilise multiple receptors for the facilitation of entry into susceptible cells ( Patel and Bergelson 2009 ; Hayes et al. 2006 ; Yamayoshi et al. 2009 ). Thus the identification and characterisation of cellular receptors plays a critical role in the understanding of EV71 pathogenesis. Scavenger receptor class B, member 2 (SCARB2) was first reported by Yamayoshi et al. ( 2009 ) to be a receptor for all EV71 strains and expressed in the sites of EV71 replication *in vivo* ( Hayes et al. 2006 ; Yamayoshi et al. 2009 ). It is composed of 478 amino acids and belongs to the CD36 family ( Yamayoshi et al. 2012 ). SCARB2 is commonly found in abundant in the lysosomal membrane of the cell and assist in the internalisation of EV71 into the host cell via clathrin mediated endocytosis ( Hussain et al. 2011 ; Lui et al. 2013 ; Yamayoshi et al. 2012 ). The presence of SCARB2 in HT29 further supports HT29 cells as a viable *in vitro* model to study EV71 pathogenesis.

Considering that different cell types have varying cellular content such as cytoskeleton and endoplasmic reticulum network which would potentially plays a role in virus replication, it is therefore the virus replication kinetics may differs from cell types to cell types. Indeed, Hussain et al. ( 2011 ) has demonstrated that the cytoskeletal system comprising of both actin and microtubules were involved endocytic kinetics ( Buss et al. 2001 ; Durrbach et al. 1996 ; Flanagan and Lin 1980 ; Hussain et al. 2011 ). The knockdown of genes involves in cytoskeleton formation such as ARPC5, ARRB1, and WASF1 and the use of drug disrupting the cytoskeleton network such as cytochalasin B, have resulted in the decrease in EV71 replication kinetics ( Hussain et al. 2011 ; Lui et al. 2013 ).

Furthermore, the incubation period for HFMD is between three to seven days ( Khong et al. 2012 ; Ooi et al. 2010 ; Ooi et al. 2009 ; Ooi et al. 2007 ). This may correspond to the number of days the virus requires to pass through the gastrointestinal tract before spreading it throughout the body Khong et al. 2012 ; Ooi et al. 2010 ; Ooi et al. 2009 ; Ooi et al. 2007 ). Therefore the *in vitro* model of human epithelial colorectal cell line (HT29) and EV71 may be more clinically relevant and mimics the mechanism of pathogenesis of EV71 closer.

## Conclusions

In conclusion, we have established the use of HT29 cells as a clinically relevant *in vitro* model of EV71 replication. We have demonstrated for the first time an increase of viral concentration in a time course of 72 hours upon infection with the use of cell viability, qPCR and western blot. In addition, this is the first report on the presence of SCARB2 on HT29 cells, an essential receptor for all EV71 strains which established HT29 cells as a viable *in vitro* model to study EV71 pathogenesis. Our study has provide valuable knowledge toward the study of EV71 pathogenesis, virus-host interaction and this could lead to future investigation for the development of antiviral therapeutics against EV71. Therapeutic agents against EV71 could be developed by potentially inhibit several key stages of the viral life cycle such as viral attachment, translation, polyprotein processing and RNA replication with the use of HT29 as an *in vitro* model for EV71 replication ( Chen et al. 2008 ).

## Methods

### Cell culture and virus propagation

Human colorectal cell line (HT29) (ATCC® catalog no. HTB-38™) was maintained in Roswell Park Memorial Institute medium (RPMI) (PAA Laboratories, Austria) supplemented with 10% (v/v) Fetal Bovine Serum (FBS) (PAA Laboratories, Austria) and 2% penicillin–streptomycin (PAA Laboratories, Austria) at 37°C with 5% CO_2_. The EV71 strain used in this study was isolated from a fatal case of HFMD during October 2000 outbreak in Singapore, Enterovirus 5865/sin/000009 strain (accession number 316321; hereby designated as Strain 41) from subgenogroup B4. The virus stock was prepared by propagation of viruses using 90% confluent HT29 cells monolayer in RPMI with 10% FBS and 2% penicillin–streptomycin at 37°C with 5% CO_2_. The virus titres were determined using 50% tissue culture infective dose (TCID_50_) per millilitre (mL) according to Reed and Muench method ( Reed and Muench 1938 ).

### Viral infection

HT29 cells were seeded at a concentration of 2 × 10^6^ cells/ml in 6-well plates and incubated for 24 h at 37°C with 5% CO_2_. Cells were washed twice with phosphate buffered saline (PBS) and infect with EV71 at multiplicity of infection (MOI) of 1 or nil respectively. Following infection for 1 h, the culture media were removed and replaced with 2 mL of fresh RPMI medium. Micrograph was then taken using phase contrast microscopy at different time points after which the cells were trypsinised and harvested at 12 h, 24 h, 48 h and 72 h to isolate RNA and proteins for qPCR reactions and western blots respectively.

### Cell viability and counts

Cell count and viability was performed on the Luna™ Automated Cell Counter system (Logos Biosystem, USA) in accordance to the manufacturer’s instructions. Briefly, the cells were trypsinised at different time points (12 h, 24 h, 48 h and 72 h). The trypsinised cells were then topped up with fresh media to a total volume of 1000 μl of media and 10 μl of this cell suspension were mixed with 10 μl of tryphan blue. 10 μl of this diluted cell suspension were then loaded onto the Luna™ counting slide for analysis.

### RNA isolation and cDNA synthesis

The total cellular RNA of HT29 cells were extracted using the miRNeasy mini kit (Qiagen, Hilden, Germany) in accordance to the manufacturer’s instructions. Briefly, the cells were lysed and homogenise using lyses solution provided (Qiagen, Hilden, Germany). Total RNA were harvested using the RNeasy spin column and wash twice before elution (Qiagen, Hilden, Germany). Harvested total RNA was quantitated using Nanodrop 100 spectrophotometer (Thermo Scientific, Waltham, USA) and 1 ng of the total RNA was then reverse transcripted using the iScript™ cDNA Synthesis Kit (Bio-Rad Laboratories, CA, USA) in accordance to the manufacturer’s instructions. Briefly, 1 ng of the extracted RNA was mixed with enzyme reverse transcriptase and buffer to a volume of 20 ul and subjected to thermal profile of 25°C for 5 m, 42°C for 30 m followed by 85°C for 5 m in accordance to the manufacturer’s instructions.

### Quantitative real time polymerase chain reaction

The EV71 specific primers targeting the conserve VP1 regions were 5′-GCTCTATAGGAGATAGTGTGAGTAGGG-3′ and the reverse primer 5′-ATGACTGCTCACCTGCGTGTT-3′ ( Tan et al. 2008a ). Primers for SCARB2 receptors were 5′- CCAATACGTCAGACAATGCC-3′ and the reverse primer 5′-ACCATTCTTGCAGATGCTGA-3′ were designed to span exon-exon boundaries. The primers for the house keeping gene actin (ACT) used were 5′- ACCAACTGGGACGACATGGAGAAA-3′ and the reverse primer 5′-TAGCACAGCCTGGATAGCAACGTA-3′. The quantitative real time polymerase chain reaction (qRT-PCR) was performed using the iQ™ SYBR® Green Super mix (Bio-Rad Laboratories, CA, USA) on the Bio-Rad CFX96™ Real-Time PCR system (Bio-Rad Laboratories, CA, USA). Briefly, 1 μl of cDNA and 1 μl of the forward and the reverse primers were added to iQ™ SYBR® Green Super mix. The reaction mix was then subjected to thermal profile of denaturation at 95°C for 10 m, followed by amplification and quantification in 40 cycles at 95°C for 10 s, 60°C for 30 s followed by 50°C for 30 s. At the end of amplification cycles, melting temperature analysis was performed by the Bio-Rad CFX96™ Real-Time PCR system (Bio-Rad Laboratories, CA, USA). Relative gene expression was quantified based on the formula: 2(^Ct of gene-Ct of ACT^).

### Western blot

Total cellular protein extraction for HT29 cells and control cells were performed using a lysis mix in mammalian cell lysis solution – CelLytic M (Sigma-Aldrich Pte Ltd, USA) in accordance with manufacturer’s instructions. Equal protein concentration (20 μg) from each samples were added into SDS PAGE, 10% Mini-PROTEAN® TGX™ (Bio-Rad Laboratories, CA, USA) and separated by electrophoresis. Separated proteins were transferred onto polyvinylidene diflouride membranes (Invitrogen, California, USA) using iBlot® Western Detection kit (Invitrogen, California, USA) in accordance to manufacturer instructions. Ponceau S staining was performed to ensure equal level of protein present in all lanes according to Romero-Calvo et al. ( 2010 ) ( Romero-Calvo et al. 2010 ). Briefly, membranes were stained with Ponceau S (Sigma-Aldrich Pte Ltd, USA) for 1 m and washed three times with water to remove stain. Western blot was then performed using Western Breeze® Chromogenic Kit–Anti-Mouse (Invitrogen, California, USA) in accordance to manufacturer instructions. Briefly, the membranes were incubated with mouse anti EV71 antibody (AbD serotech, Oxford, UK) or anti Tubulin antibody (Santa Cruz Biotechnology inc, California, USA) respectively in shaker for an hour. The membranes were washed three times before and incubating with secondary antibodies (Invitrogen, California, USA) for 30 m. The membranes were washed three times and incubated with chromogen substrate till purple bands were developed in 1 h. The membranes were left to air dry then placed into the densitometer and scanned using the Quantity One software (Bio-Rad Laboratories, CA, USA). The picture was analysed using Image J (National Institutes of Health, USA).

### Statistical analysis

All statistical analysis was performed on Graph Pad Prism Version 6.0c (Graph Pad Software, USA). Student *t* test was used to compare two groups. *p* values of < 0.05 were considered statistically significant.

## Electronic supplementary material

Additional file 1: Table S1: Proposed timeline of EV71 replication kinetics in HT29 cells. (DOCX 14 KB)
