# Supplementary material for: Characterisation of enterovirus 71 replication kinetics in human colorectal cell line, HT29
Source: Springerplus. 2013 Jun 18;2:267. doi: 10.1186/2193-1801-2-267 (PMC3696168; doi:10.1186/2193-1801-2-267)
Supplement: Supplementary file 1 — Additional file 1: Table S1: Proposed timeline of EV71 replication kinetics in HT29 cells. (DOCX 14 KB) [file 40064_2013_339_MOESM1_ESM.docx]

| -1 | 0 | 12 | 24 | 48 | 72 |
| --- | --- | --- | --- | --- | --- |
| Attachment and entry |  |  |  |  |  |
|  | Uncoating | |  |  |  |
|  |  | RNA replication | | | |
|  |  |  | Protein synthesis | | |
|  |  |  |  | Package | |
|  |  |  |  | Secretion | |

**Additional file 1: Table S1. Proposed timeline of EV71 replication kinetics in HT29 cells.**

HT29 was co-cultured with EV71 at multiplicity of infection (MOI) of 1 for 1h where the EV71 attaches and gain entry into the host cell. Thereafter the culture media were replaced with 2 mL of fresh RPMI medium where the EV71 undergo uncoating and replication of their genomic RNA. Viral RNA and protein was first detected at approximately 12 hpi and 48hpi present throughout the 72h time course. Finally EV71 undergo packaging and secrete newly synthesised viruses to infect new cells evidenced by the cell viability at 48hpi and 72hpi.
